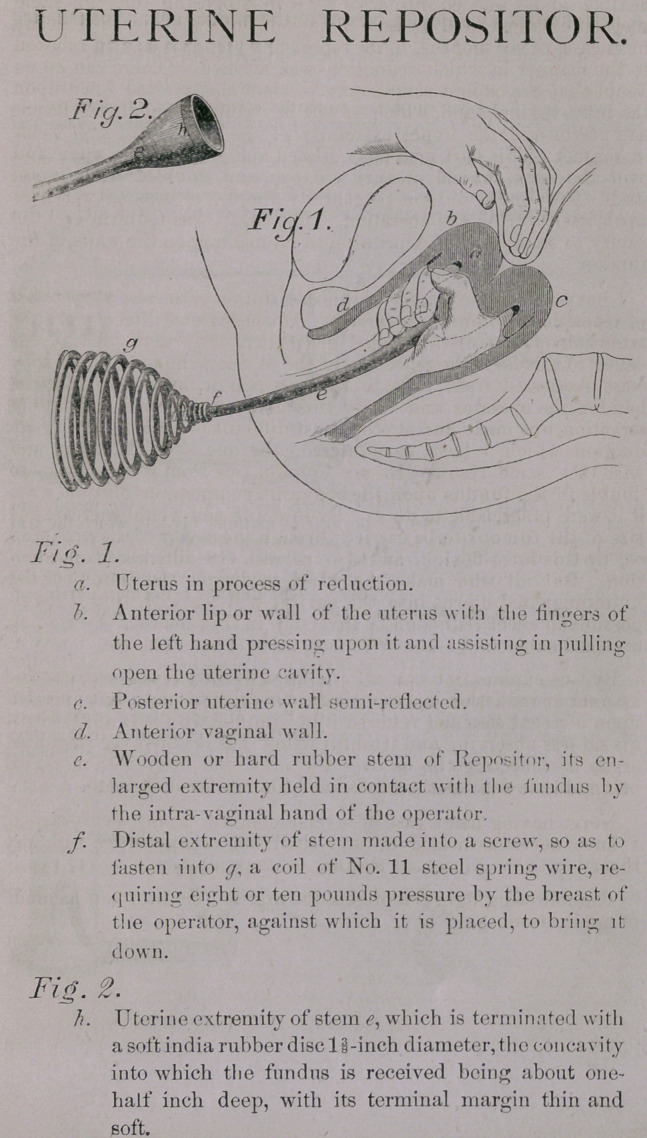# Report of Two Cases of Inversion of the Uterus, with Remarks and a Description of the Uterine Repositor

**Published:** 1872-05

**Authors:** James P. White

**Affiliations:** Professor of Obstetrics in the Medical Department of the University of Buffalo


### Miscellaneous.

(From American Journal of the Medical Sciences.)

### Report of Two Cases of Inversion of the Uterus, with Remarks and a Description of the Uterine Repositor.

By James P. White, M. D.,

Professor of Obstetrics in the Medical Department of the University of Buffalo. (Read before the Medical Society of the State of New York. February 6, 1872.)

The *Eighth Case* of inversion of the uterus, which has been treated by me, occurred near Ithaca, Tompkins County, New York.

The following is an extract from a letter received from Dr. H. B. Chase, of Jacksonville, who was then in attendance upon the patient:—

"The case is as follows; Mrs. R., æt. 27 years, of a good constitution, was delivered on the morning of the 25th January, of her first child, after a tedious labour of eighteen hours, when her medical attendant discovered that the placenta was adherent. Severe hemorrhage came on when the doctor separated the placenta from the walls of the uterus and withdrew it with his hand. Immediately (so he says) he discovered a large solid tumour in the vagina, accompanied by still more profuse hemorrhage.

"Four days after the accident I was called to see the patient. On examination I found the uterus completely inverted. The os uteri could be felt at the superior extremity of the tumour. The uterus, at that time, hard and unyielding and filling the vagina. I made an effort to reduce it, but failed. I felt quite sure that reduction was impossible at that time. And now, doctor, the question I would ask is; Can anything be done to restore this poor woman to health and usefulness again?"

My reply to Dr. Chase's inquiry held out the hope that it could, by suitable manipulation, be restored. Accordingly, in compliance with an invitation contained in a subsequent letter, I visited the patient on the 27th of February, not quite five weeks after the accident.

I found her very exsanguine and feeble, constantly losing blood, and at times bleeding profusely. The uterus had not completed its involution, though it was nearly as small and firm as in the normal condition of the organ in the multipara in its unimpregnated state. The perineum had been lacerated during the labour, and the hand could be easily introduced into the vagina and up to the neck of the uterus. The inversion was found to be complete, although the uterus did not protrude beyond the vulva.

The bowels had been, by my direction, freely evacuated the same morning, and, after drawing off the urine, I proceeded to reposit the organ, in the presence of Drs. Chase, Lewis, White, and Carrington and Mr. George Rightmire, medical student, who has furnished a partial report of the case in the *Buffalo Medical Journal* for April 1871. Requesting one of the gentlemen to take charge of the anæsthetic, whilst two others were seated beside the bed, each holding in his lap a foot, supporting with one hand the knee upon his side and holding firmly the patient's hand upon the same side, with her hips brought to the edge of the bed, I placed myself upon my knees and commenced the operation. Carrying my right hand into the vagina, I seized the entire organ and manipulated and compressed it firmly for a short time, to expel the blood and render the tissue flexible. This pressure was continued, with the hand within the vagina, during the entire process. I now commenced pushing upon the fundus, with a large rectal bougie, alternating with the thumb and fingers of the left hand and thus put the vagina upon the stretch. This process, through the attachment of the vagina to the inverted uterus, pulled open the os and reflected it upon the neck. Thus it will be perceived, as I have heretofore described, the process of restoration was from neck to fundus. The fundus was at no time perceptibly dimpled or doubled in upon itself. By continuing this process steadily and gently, occasionally placing the fingers of the left hand over the hypogastrium and through the thin walls of the abdomen, inserting them into the opening os and making traction and pressure, I found the organ passing up through the neck and assuming its normal position. At the expiration of twenty-three minutes from the time of commencing the operation, the uterus was completely restored. The loss of blood was small, and the only noteworthy peculiarity in the case was the doughy *feel* of the organ in this stage of involution. It did not possess the firmness of normal uterine tissue in the unimpregnated state, nor the muscular flexibility of the recently delivered organ; but had a doughy feel like an organ undergoing fatty degeneration. Indeed I am induced to suspect that, at this period after delivery, the uterus cannot be subjected to manipulations without danger of laceration, which would be perfectly safe at a later period, after complete involution had taken place. It is the only case which has fallen under my observation between the third and the eighth week, and the sensations occasioned by pressure and other manipulations were such as to excite apprehension lest the tissues would yield under the fingers.

The patient soon recovered from the effects of the anæsthetic, and I left her, a few hours after, sleeping comfortably under the influence of an anodyne. Since that time she has, under the care of her attending physician, Dr. Chase, gradually convalesced, and is now in "good health."

Case IX.—The following history is taken from notes furnished by J. W. Stewart, M. D., the attending physician:—

"Mrs. E. A., of Port Dover, Canada, æt. 34, was delivered of her second child on the 7th of October 1870. The case for the first seven days I had no personal observation of, but will give a brief history as I learned it from the patient herself and her friends who were attending her, the correctness of which I do not doubt.

"The labour was comparatively an easy one, lasting but three or four hours, and having no pains as usual for a few minutes after the birth of the child. The medical attendant, finding that the placenta was not expelled, made an examination, and said 'it was grown to the womb.' He caught hold of the cord, giving it two or three sharp jerks, and the after-birth came away. The patient, immediately after its expulsion, complained of violent and constant pain, and says she experienced a sensation as if 'all her insides had dropped down.' In a few minutes she began to sink, being bathed in cold perspiration, pulse running very rapidly. Her friends, being alarmed, asked the doctor what was the matter? He replied she was dying. After a time she rallied, but remained in excruciating agony up to the time I saw her—the eighth day after confinement. At my first visit I found her in the following condition: Tongue deeply furred; very irritable and feverish; pulse 155 to 170, very angular and weak; abdomen much swollen and so tender she could not bear the clothes to touch her. In a word, I found her labouring under a violent attack of metritis, the inflammatory action extending over the whole surface of the peritoneum. I learned that these symptoms had appeared three days previously, hourly growing more intense. I removed her bandage immediately, and applied turpentine stupes and warm fomentations, giving her large and repeated doses of opium.

"By my advice Dr. C. W. Coventer was called in the same evening and the next day. She described to us so accurately the symptoms of inversion of the uterus, taken in connection with the history of the case and her strange feelings, that we suspected the true state of affairs, and suggested an examination. This she positively refused, saying she 'was so weak and tender it would kill her,' and we were compelled to postpone it till the next day. The next morning I found her better, and the whole surface of the abdomen terribly blistered from the free use of turpentine. The inflammation and swelling were subsiding rapidly, and I insisted on making an examination; when I found the uterus inverted, and almost protruding from the vagina.

"Continued the warm fomentations, keeping her thoroughly under the influence of opium, with quinia, beef-tea, &c. &c.

"In a few days she improved wonderfully, and in two weeks we considered her strong enough to bear the operation, and attempted its reduction. We first brought her under the influence of chloroform, and then introduced the hand into the vagina, trying to reduce it by a kneading process with constant pressure. We succeeded so far, after two hours' constant labour, as to get the fundus even with, if not a little inside of, the os. We tried to keep what we had gained by inserting and inflating gutta-percha balls, which we had procured for the purpose. Next day she appeared to suffer very little from the operation; but the India-rubber balls did not come up to our expectations, so the womb had dropped back nearly into its former position.

"About eight weeks after, we made a second attempt, but the result was less favourable than the first. I forgot to mention that, about midway between the first and second operations, she had another violent attack of inflammation without apparent cause."

Upon receiving an invitation from Dr. Stewart, I visited the patient on the 11th of March, 1871, nearly six months after the inversion. I found the woman very prostrate and anæmic, and suffering from constant copious leucorrhœal discharges, and frequent hemorrhages. The uterus was completely inverted, with its fundus just within the vulva, and having fully undergone the process of involution. It was now as hard and firm as in the normal condition in the multiparæ. The bowels, by my direction, had been freely evacuated before my arrival, and I preceeded at once to make an effort to reposit it.

Placing her in the same position as heretofore described—upon the side of the bed, with her feet supported by Drs. Coventer and Salmon, I assumed my usual position upon my knees. Dr.Stewart, her family physician, was charged with the administration of the chloroform, and requested to produce profound anæsthesia. The first few minutes were chiefly occupied in compressing and moulding the uterus, rendering its walls more flexible, and in gently pressing up the organ so as to put the os upon the stretch and commence its dilatation. At this time the *uterine repositor* was introduced into the vagina, and its disk placed in contract with the fundus, and firmly held there by the intra vaginal hand. The outer end of the instrument was placed against my breast, on the same level with the uterus. By means of the large circular spring, it readily kept its place, and left the other hand free to be used above the pubes to assist in forcing open the dilating os, which could be plainly felt through the abdominal walls. The spring at the outer end of the instrument enabled me, without danger of lacerating the tissues, to keep up a constant gentle pressure upon the fundus, and by leaning forward, to increase this pressure intermittingly. The force was exerted more directly upon the fundus by means of the repositor than was possible by the thumb or fingers, or by the round end of the large bougie. 'Pressure made through this spring is made more continuous than when made by the hand, and far less likely to lacerate the tissues. It was the first time I had used it, and I was delighted to find that it gave me a third hand, *which did not become fatigued,* and permitted me to use the left hand in manipulating the os in the hypogastrium; while the right hand easily held the instrument in contact with the fundus, and firmly grasped that part of the uterus not yet reflected, and remaining in the vagina. At the expiration of an hour and ten minutes, considerable progress had been made in reflecting the os down upon the body of the organ, when the patient seemed in some danger from the chloroform, and the process was for a short time suspended. The windows were thrown open, a little brandy was given by Dr. Hayes, who was present, and in a short time it was again deemed safe to resume the operation. Remaining continuously in the same position, I resumed pressure upon the repositor, holding, with my left hand, the anterior reflecting surface of the uterus which extended further and further up into the abdomen. In one hour and twenty-three minutes from the time of taking my position, I had the pleasure of announcing that the organ was completely restored. As my associates had previously made much longer efforts, under circumstances much more favourable, they were at first incredulous, but soon verified my assertion instituting, at my request, a careful examination.

Placing the patient comfortably in bed, and giving a full dose of morphia, she slept well that night, and I left her next morning feeling quite comfortable, and delighted with the prospect of being again restored to health and strength.

Nothing remarkable occurred during her convalescence, which, though slow, was uninterrupted, "riding out" during the month of April; and in the course of the summer, she regained, I understand from Dr. Stewart, her "usual health."

*Remarks.*—These two cases complete a series of nine cases of inversion, varying in duration from a few minutes to fifteen years after its occurrence, which have been reduced, by the writer, by manipulation of a single effort. The first case was reported to the Buffalo Medical Society in February, 1856, and was of only eight days' standing. After giving a full account of the case, which was published in the *Buffalo Medical Journal* of March, 1856, the writer concludes with the following remarks:—

"This case is regarded as interesting in many respects. It will encourage the growing belief among accoucheurs that reduction may be undertaken with reasonable hope of success, at a period much later than most writers have heretofore advertised."

In the same article, alluding to a case of fourteen days standing which had fallen under my observation in 1842, and in which no attempt at restoration was made, it is added: "With my present views upon this subject, I should abandon such a case as hopeless only after a long effort at reposition."

Fully convinced, from the result of the efforts made in this instance, eight days after inversion, of the feasibility of restoring the uterus in many cases heretofore considered irreducible, I did not meet with an opportunity of putting my convictions again to the test until the 12th of March, 1858, when I visited Mrs. M., near Hornellsville, who had been confined on the 22d of September previous, almost six months before; and after about one hour's continuous effort, succeeded in repositing the inverted organ. A full account of this case, with plates, and a description ot the manner in which reduction was accomplished, was published in the July number of the *American Journal of the Medical Sciences,* for 1858. Permit me here to crave indulgence for one word in relation to priority in this operation. It is true, that Tyler Smith published his case April 24th, 1858, in the *London Medical Times and Gazette,* forty-two days after my operation in Hornellsville, and more than two months before the report of the case made its appearance, in the *American Journal of the Medical Sciences.* But I submit, that, more than two years before, I had taken the initiative, and published my views and hopes. And I also insist that his subsequent success in April, 1858, could hardly have been known in this country on the 12th of March preceding, which is the date of my second operation. Upon this point I trust I shall be pardoned for gratefully quoting the following passage from the able article on this subject from the impartial and distinguished Professor of Obstetrics in the University of Louisville.

"Before I proceed to do this [give an account of the method of manipulation pursued by Dr. White], it is meet that I should pay a tribute of honour to our countryman, Professor James P. White, who has not received the credit he so well deserves, for his leadership in the revolution of gynæcological practice, which he inaugurated. Even a superficial reading of his report[1] must satisfy any one that he regarded himself as a pioneer in his attempt to reduce, by the taxis, chronic inversion of the uterus; and that he was wholly unconscious that such a surgical feat had been performed either at home or abroad. Neither had it been so much as thought of at home, much less performed, nor had it been performed abroad in such a way as to attract professional attention or inspire confidence in it, until Dr. Tyler Smith, of London, published a case of twelve years' duration in the *Medical Times and Gazette*, April 24, 1858, which of course could not have been known to Dr. White, whose case was successfully treated on the 12th of March immediately preceding, though not published until July following, the earliest time that it could be published in a quarterly journal."[2]

On the 24th of August following, in the presence of Professors Austin Flint, Sen. and Jr., and Professors Thomas F. Rochester and Mason, I reduced an inverted uterus of over fifteen years' duration. This was accomplished in about fifty minutes and with less difficulty than those of six months. It should be stated, that the patient died on the sixteenth day after the operation, though it does not follow that her death was a consequence fairly chargeable to it.

An account of this case, as reported to the Buffalo Medical Association, will be found in the *Buffalo Medical Journal* for October, 1858, and from which the following paragraphs are cited:—

"She improved rapidly, with no untoward symptoms during the succeeding eight days, and at the end of that time considered herself perfectly well. On the morning of the ninth day, however, after returning from breakfast, she imprudently strained considerably at stool, and was suddenly seized with violent pains in the abdomen. She was at the same time startled by the coming in of a friend whilst she was on the vessel. The mother and husband had left her a day or two before, considering her perfectly well. She immediately went to bed, sent for me, and died on the seventh day after of peritonitis."

In commenting upon the report of this case in the Association, Professor Austin Flint, sen., holds the following language:—

"I was present at the reduction of the uterus by Professor White, and also at the post-mortem examination. The reduction was effected with great ease, and under the moderate use of chloroform. He (Professor Flint) regrets, equally with Dr. White, the unfortunate accident which resulted in the death of the patient. The connection between the peritonitis and the operation did not seem to him to be very close; otherwise it would have supervened sooner. He did not think that it at all invalidates the success or propriety of the operation. The result will, perhaps, be thought to have more connection with the

[1] Alluding, doubtless, to the case of 1858, and never having had his attention called to the one of January, 1856, reported in the Buffalo Journal more than two years before.

[2] Thoughts on Chronic Inversion of the Uterus, specially with reference to Gastrotomy, as a substitute for Amputation of the Uterus. By Henry Miller M. D. Richmond and Louisville Medical Journal, April, 1870, p. 14.

operation from an ordinary perusal of the case, than really exists. But a careful review of all the circumstances seems to show that the peritonitis was merely an unfortunate accident," &c. &c.

Professor Rochester said, "there were some points which had not been sufficiently dwelt upon by Dr. White or Dr. Flint. The appearance of the uterus, on examination after death, plainly indicated that no undue amount of force had been used in the operation. . . . In regard to the occurrence of peritonitis, he thought that it was a most unfortunate accident. Three-fourths of the cases which he had seen occurred at this season of the year. There was no doubt but that the exhausting nature of the difficulty under which the patient had laboured for so long, made the system more susceptible to the disease, and much less able to resist it. There was nothing in the appearance of the parts which would lead to the suspicion that the peritonitis commenced on the uterus; that it appeared to be a simple case of the disease. The greatest pain was referred to the epigastric region, and it was probable that this was its origin."

This case, of fifteen years, was much more easily reduced han the ninth case, just reported, and I am convinced that the opinion long since published by the writer that the difficulty of reduction is "as great immediately after complete involution, as at any subsequent period, however remote," will be found to be correct. I fail also to perceive why the *dangers* should be greater in consequence of delay, and hence my anxiety to prevent any erroneous conclusions upon that point which might be hastily drawn from the fact that this case terminated fatally, whilst those of six months recovered. The uterus, it is well established, completes the process of involution in from eight to twelve weeks. Judging from the cases of eight days, twenty-one days, and thirty-five days, and the eighth case just reported, it is, in my opinion, far more likely that it will be found by careful subsequent observation that the tissues are more susceptible to laceration from the necessary manipulations, during the latter part of the period of involution, than at any other time. As intimated in the report of this, the eighth case, which was between five or six weeks after labour, the impression in handling the uterus was that it would be easily torn. It is, during this change, neither muscular and flexible like the recently delivered uterus; nor firm and elastic like the unimpregnated organ. It will, I suspect, be found safer, in view of this condition, after about the twentieth day, to wait for the completion of this process, notwithstanding the increased difficulties of the operation occasioned by such delay.[1]

Observation and careful reflection—based upon the cases which have been committed to my charge, now numbering nine in all, varying, as already stated, in duration from a few minutes to fifteen years—confirm me in the opinion expressed in 1858. Indeed, my convictions are very decided, and I am incredulous as to the neces-

[1] Pages 102 and 103, Lectures by Priestley, on the subject of the involution of the uterine parietes. says: "With the advance of the fatty transformations the uterus becomes, in a corresponding degree, friable, and continues so until it has completely returned to its usual condition. I have occasionally seen at the post mortem examination of women. who had previously borne children, the uterine tissue affected by fatty degenerations, and so soft and friable that a sound passed into the uterine cavity might be readily pushed through the uterine walls.

sity of ever resorting to amputation, or the still more objectionable operation of gastrotomy. True, it may not always be reduced at a single sitting, as in all which I have encountered, but by means of the repositor, uniform and gentle pressure can be continued until the os is fully dilated, and the fundus pushed up through it. The insurmountable difficulty heretofore has been supposed to consist in our inability to maintain uniform and persistent pressure for a sufficient length of time. The hand soon became fatigued, and another hand, even of the same individual, could not be substituted without losing a part of what had been gained. This loss was increased when the hand of a fellow practitioner was introduced to continue the operation. No doubt great physical endurance, being able to maintain one position for a great length of time, has been an essential element in success. The various substitutes which have heretofore been resorted to for continuing pressure, where the operator became exhausted, have utterly failed. The elastic bags, mentioned in the last case, used by Dr. Stewart, are a fair illustration of their inefficiency. They press more upon the large surfaces anteriorly and posteriorly situated, and with which they are in contact, than they do upon the fundus, which has no firm ossific base of support, as have the rectum and bladder. The uterus ascends very soon, owing to the yielding nature of the vagina, and escapes from the reach of the distended vaginal bags.

The repositor, when it is deemed better to proceed in a more gradual manner, or when it may be found impossibte to reduce it at a single effort, can be received with the uterus into a large cylindrical speculum, and by means of a T-bandage, can be made to press directly upon the fundus until the os is gradually dilated and all resistance overcome. By means of the large spring at the outer extremity the amount of pressure can be graduated to an ounce. The disk will follow up the fundus by means of this continuous elastic pressure until it disappears in the os and neck. Any intelligent assistant can be trusted to increase or diminish the pressure during the absence of the practitioner, as the exigencies of the case may demand. The pressure which the repositor can be made to exert is equally as gentle and far more persistent than the tent of sponge or sea-tangle, or the wedges of Barnes, when applied to dilate the os for the purpose of examining the cavity, when the uterus is in its normal position. These are the views expressed fourteen years since, in the article in the *American Journal of the Medical Sciences*, already alluded to. The following passage occurs :—

"We are all aware that the os and neck of the uterus may, by sponge tents and other mechanical contrivances, be widely dilated when the womb is in its natural position and of ordinary size. This dilatation is frequently made by those who are extensively engaged in the treatment of uterine affections for the purpose of perfecting diagnosis or instituting treatment. Of the susceptibility of these parts to be thus dilated, and that, too, without much risk of injury to the tissues involved or the general health, no one at all familiar with the subject will deny. What is the relation of parts in inversio uteri? Is not the vaginal sheath, which in the normal arrangements of parts was attached to the outside of the neck of the uterus, now, in the inverted state, firmly attached *within* the cavity of the canal of the neck of the uterus just at its orifice? The vaginal canal is securely attached at its lower extremity at or near the outlet of the pelvis, and, whilst it is very elastic, is less yielding in its longitudinal than transverse diameter, and not easily lacerated or detached from its connections, unless irregularly pressed as by a pointed instrument. Force or pressure, applied to the fundus of the inverted uterus, is resisted by the upper extremity of the vagina, which is now fastened upon the inside of the neck. The lower extremity of the vagina, being firmly attached, cannot yield, and the inevitable result must be the pulling open the mouth of the uterus, unless the tissues are lacerated before that part dilates. Can there be any greater difficulty or danger in *pulling* open this outlet than in *pressing* it open if performed with the same gentleness?

"The uterus and vagina in complete inversion represent a continuous bag or sac, doubled or reflected upon itself, with the open extremity of the sac securely fixed Pressure upon the closed end of the bag will, it is plainly perceived, under such circumstances, result in straightening the bag by completely turning it the other side out. So with the parts concerned in inversion. Pressure upon the fundus, if well directed, pulls open, first its mouth, then its neck, and finally, if persevered in, doubles the body upon itself also, and carries the fundus through the os and neck and body to its normal position.

"Does any uterine pathologist believe it would be impossible safely to dilate the os and bring down the fundus of the uterus—completely inverting the organ—if carefully and perseveringly undertaken? If affirmatively answered, then why may we not pull open the neck by means of the vagina in the same gentle manner as we would press it open when in a normal position, and thus carry the fundus up through it by means of pressure upon that part when it is in a downward direction? Perhaps I may be too sanguine, but I am inclined to believe that well-directed pressure upon the fundus, if continued long enough, will in all cases, where there are no adhesions, result in restoration or reposition, no matter how much time may have elapsed since inversion occurred."

Subsequent experience has but served to confirm the views then advanced. With the repositor, as before described, pressure is brought directly upon the fundus and maintained for any length of time desired and with such an amount of force as shall be deemed entirely safe. The uterus can only escape in an upward direction, and restoration must follow. Nor will it be more painful when thus dilated than when the os is dilated by tents. I am of opinion that all cases of inversion *can* be reduced, without qualification as to their duration.

This opinion does not, however, seem to have been generally adopted by writers upon the subject, since these views were published, and hence it is deemed important to urge them upon the consideration of gynæcologists.

It is true, there are a few exceptions to this lack of faith. In an able "Report on Inversion of the Uterus," made to the New York State Medical Society, by appointment by Prof. J. V. P. Quackenbush, at its annual meeting in February, 1859, we find the following emphatic endorsement of the feasibility of reduction. After giving a very clear account of his theory of the causes of this accident, he says;—

"But whatever may be the manner of its occurence; the proper treatment is what most demands our attention. And what is the proper treatment of inversion of the uterus? I answer, reduction. If the case be recent, reduction; if of twelve days' duration, reduction; if of twelve years' duration, reduction! At any time, and under all circumstances, reduction must be resorted to, and very few cases will be found which are not irreducible. I lay much stress upon this subject, for I do not think it well understood" . . . Again, after referring to the opinion of Meigs when he says, "you have no art or skill or no power equal to the performance of such a miracle of surgery as that" (the reduction of a case of inversion of six months' duration), Prof. Q. adds, "gentlemen, this miracle has been done, and can be done, and should be done, and with proper management no case need be abandoned."

Notwithstanding this unhesitating affirmation of the doctrine of the feasibility of reduction in all cases of chronic inversion, which I had enunciated nearly two years previously, we find men in this state holding to the old doctrine that reduction is exceptional, and not by any means to be expected. The late Prof. Bedford, in his *Principles and Practice of Obstetrics,* Fourth Edition, 1868, uses the following language:—

Should every effort *fail*—and such, in the most skilful hands, will not *unfrequently* be the case—care should be taken to return, if possible, the tumour within the vagina, and retain it *in situ* by the India-rubber pessary, &c.&c.&c.

Nor is this incredulity, or want of progress, confined to this country. In England, where Tyler Smith had reduced a case of twelve years' standing, and published it in April 1858, expressing the opinion "that no case, with proper and prompt management, could be considered irreducible within a reasonable time after the accident," we find Dr. Meadows, in his *Manual of Midwifery,* published very recently, using the following language; "When the case has gone on unrelieved for many weeks, or even years, though attempts should always be made to secure reduction, &c. &c., yet the chances are *very greatly against success,*" &c. &c.

Again, Courty, in his work published in 1866, in Paris, and who may be considered a fair exponent of the views in his own country, refers to reduction as being exceedingly doubtful. Indeed, he describes a method of reduction of his own which his reviewer, in the *American Journal of the Medical Sciences,* thinks "especially worthy of consideration and trial." This consists in passing two fingers into the rectum and by depressing them into the anterior wall of the rectum, hooking them into the ligament on each side of the neck, and thus holding it down whilst, with the other hand, pressure is made upon the inverted portion of the organ, &c. &c. Again, he adds: "If the reduction is impossible, it is necessary to resort to palliative treatment or to *extirpation* of the uterus for its radical cure."[1] Nor are our German friends more hopeful. In a

---

[1] Page 80, Paris edition, 1866.

work by Dillenberger, republished in London, in 1871, he says: "Chronic inversion is held to require palliation; if that is vain, removal by ligature or excision."

Whence this want of confidence? The cases of reduction are now so numerous, and the operation of such demonstrable character, as not to admit of doubt.

I am utterly unable to account for this unbelief. Mere duration, I must repeat, cannot militate against success after the first three or four months have elapsed. And I am entirely incredulous as to there being such adhesions between the walls of the uterus as to withstand long-continued gentle pressure. Force may be as gradually applied by means of the repositor and speculum, as the operator may deem necessary for the safety of the patient. When the os has been thus carefully dilated, the hand of the operator, or, if the fundus has passed up into the neck, a large rectal bougie, can be called in requisition to complete the operation. Indeed, I indulge the hope that the day is not distant when all cases of inversion of the uterus will be regarded by all intelligent practitioners as amenable to treatment.

Perhaps something should be said here in relation to the manner in which reduction is accomplished. There seems great and unnecessary ambiguity and confusion on this subject. In all recent cases the fundus can be pressed into the body and neck, or "dimpled," as it is termed, by pressure upon the most depending part. In this manner, I am certain, the two cases which were reposited immediately after delivery and, to some extent, the one of eight days' duration, were carried up. In the case of Dr. Lockwood, in 1861, the womb had been inverted for about forty minutes when I arrived. Administering some paregoric and brandy to restore her from the collapse, I seized the uterus, pulled off the partly adherent placenta, and passed my hand, with the fundus before it, up into the cavity of the abdomen with scarcely more difficulty than would be encountered in inverting a wet bladder. Retaining my hand there for a short time, contraction soon came on under the influence of the ergot and stimulants which were given, and the patient [Mrs. W.] made a good recovery, and has since borne children. So with the one at eight days; in the report, it is stated, "having succeeded in 'dimpling' the fundus, pressure was maintained with the thumb at that point until the hand became nearly powerless. To preserve this depression whilst the muscles of the hand were permitted to rest, a rectal bougie, about twelve inches in length and one in diameter, was carried along in its place, fixed in the dimple, and pressure unintermittingly continued through it, by the left hand outside the vulva. Gradually the concavity of the fundus was found to be deeper until it finally became completely restored." This is doubtless the manner in which reduction takes place in all recent cases, whilst the organ is large and the walls flexible, and I was led into error by it in supposing that this same method obtained in all cases. This delusion was dispelled in my next case, of six months, in the report of which it is said in relation to the manner in which reduction was affected: "There can be no doubt that the os first commenced to yield and pressed down upon the intra-vaginal band, which, it will be recollected, inclosed the entire uterus and the upper extremity of the bougie and kept them in contact. This part gradually dilated and passed down upon and over the neck, which in turn dilated, and doubled down upon itself. The fundus did not perceptibly dimple, or was not reflected upon itself during the operation. The organ was too firm and the cavity to small for any depression to be made upon the walls of the fundus."

I have nothing to change in this description of the *modus operandi* of reposition in chronic inversion. In the case of fifteen years the attention of the distinguished gentlemen who were present was called to this fact [the reflection of the os upon the neck], and they were requested from time to time to pass a finger up beside the hand of the operator and verify this point. The same careful observation was made during the most difficult of all the cases of reduction which I have encountered, the one at Port Dover, and with the same result. In my opinion, it would be unwise to double in the fundus upon the body after complete involution, even if it were practicable to do so. By so doing you would increase the size of the tumour to be carried through the cavity of the neck and os, by this intro-flexion, and also increase the difficulties of reduction. But all who make the effort to dimple the fundus of the unimpregnated uterus into the cavity, will perceive that it is impossible to effect reduction or inversion in this manner, even upon the dead subject.

By this explanation can all the apparent discrepancies as to the manner or reduction be reconciled. One practitioner has operated upon a recent case and restored it by dimpling in the fundus, whilst his no less observing and truthful fellow has restored the involuted organ by reflecting the os over the neck and body without at all being able to depress the fundus, and hence the difference.

Never having had a case of inversion occur in my own practice, I have never witnessed it, and shall not occupy space with any theory as to the cause and manner of its production.

The following figures represent the uterine repositor and its mode of application:—

### UTERINE REPOSITOR.

## Figures and Tables

**Fig. 1. Fig. 2. f1:**